# Supplementary figures and images for: Protein Phosphorylation Orchestrates Acclimations of Arabidopsis Plants to Environmental pH
Source: Mol Cell Proteomics. 2023 Nov 23;23(1):100685. doi: 10.1016/j.mcpro.2023.100685 (PMC10837763; doi:10.1016/j.mcpro.2023.100685)

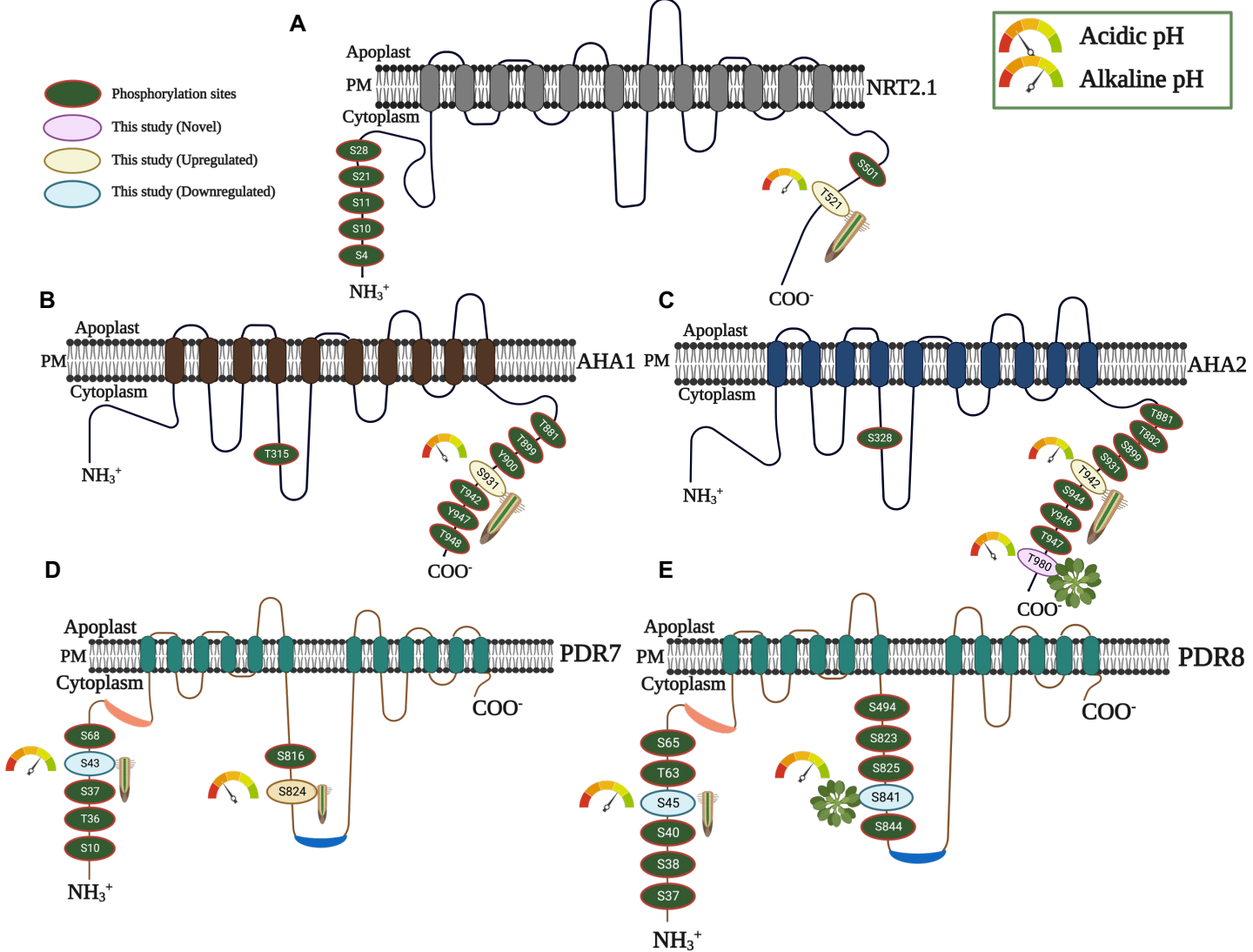

Supplementary figure 1.

Supplement: Supplementary Figure S1 [file mmc1.pdf]
