# Supplementary material for: Protein Phosphorylation Orchestrates Acclimations of Arabidopsis Plants to Environmental pH
Source: Mol Cell Proteomics. 2023 Nov 23;23(1):100685. doi: 10.1016/j.mcpro.2023.100685 (PMC10837763; doi:10.1016/j.mcpro.2023.100685)
Supplement: Supplementary Figure S2 [file mmc2.pdf]

A

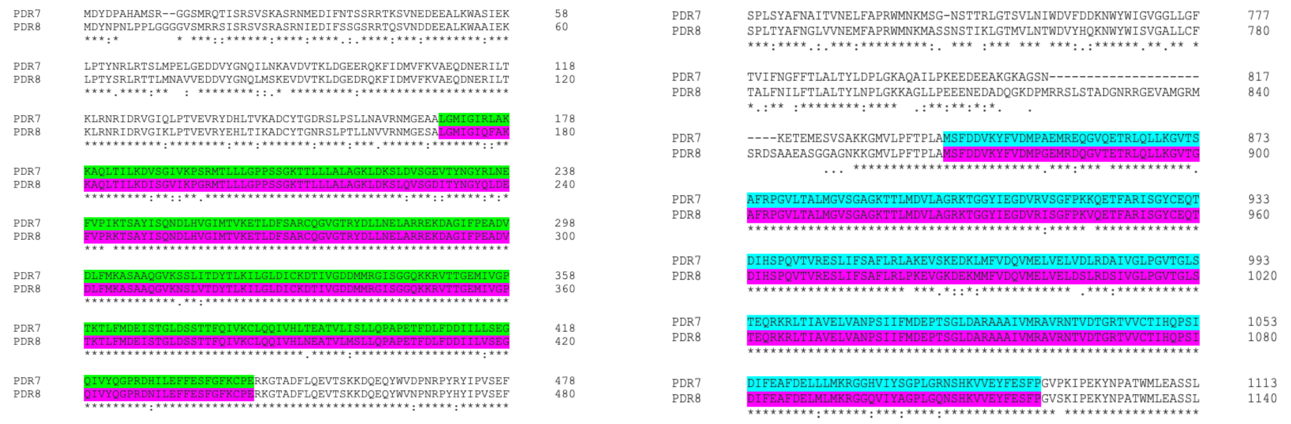

B

NBD - nucleotide binding domains

- PDR7 and PDR8 share 83% protein sequence homology -

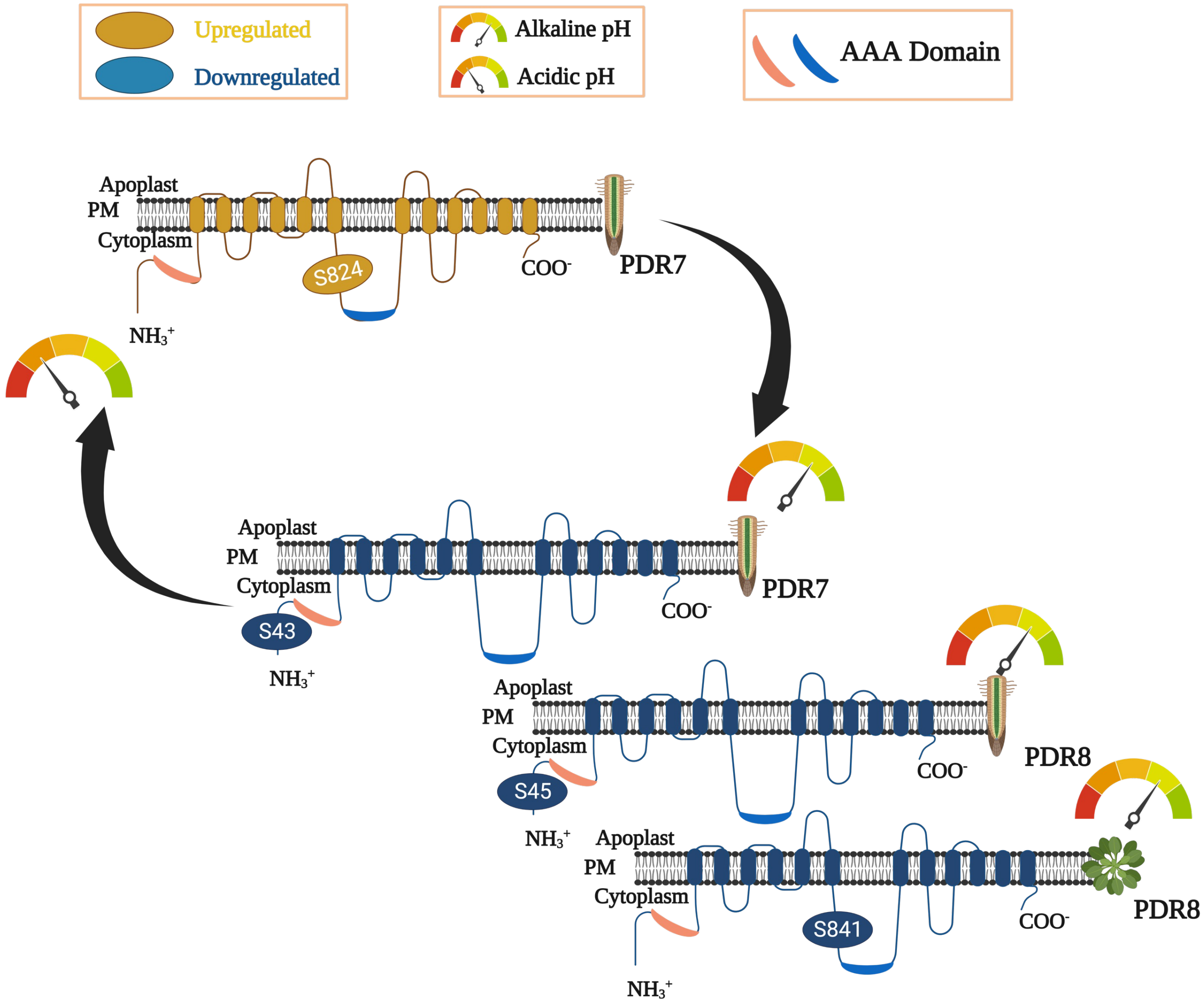

Supplementary figure 2.
