# Supplementary material for: Protein Phosphorylation Orchestrates Acclimations of Arabidopsis Plants to Environmental pH
Source: Mol Cell Proteomics. 2023 Nov 23;23(1):100685. doi: 10.1016/j.mcpro.2023.100685 (PMC10837763; doi:10.1016/j.mcpro.2023.100685)
Supplement: Supplemental Table S1 [file mmc5.docx]

| **Accession** | **Treatment** | **Tissue** | **Location SUBAcon** | **Transporter** | **Gene** | **Phosphorylation site** | **Phosphorylation (%) Probability** | **Reference/ other sites** |
| --- | --- | --- | --- | --- | --- | --- | --- | --- |
| AT1G15210 | DAPP pH_4.5 | Root | PM | **Drug** | PDR7 (ABCG35) | S824 | 100 | **(91)** |
| AT2G18960 | DAPP pH_4.5 | Root | PM | **Proton** | AHA1,OST2, PMA, H(+)-ATPase1 | S931 | 100 | **(92)** |
| AT1G18880 | DAPP pH_7.5 | Root | PM | **Nitrate** | NRT1.9 | S586 | 100 | **(93-95)** |
| AT2G32270 | DAPP pH_7.5 | Root | Extracellular | **Zinc** | ZIP3 | S164 | 100 | **(96), S163** |
| AT4G30190 | DAPP pH_7.5 | Root | PM | **Proton** | AHA2, PMA2, H(+)-ATPase 2 | T942 | 100 | **(94, 97-100)** |
| AT1G08090 | DAPP pH_7.5 | Root | PM | **Nitrate** | NRT2.1 | T521 | 100 | **(40, 94)** |
| AT5G24290 | DAPP pH_7.5 | Root | Vacuole | **Iron** | Vacuolar iron transporter (VIT) family protein | S158 | 100 | **(94, 95)** |
| AT5G24030 | DAPP pH_7.5 | Root | PM | **Nitrate** | SLAH3, SLAC1 homologue 3 | S601 | 99.8 | **(43, 44)** |
| AT1G64780 | DAPP pH_7.5 | Root | PM | **Ammonium** | AMT1;2 | T472 | 100 | **(45, 91, 101)** |
| AT4G27870 | DAPP pH_7.5 | Root | Vacuole | **Iron** | Vacuolar iron transporter (VIT) family protein | S384 | 100 | **(93), 364** |
| AT1G59870 | DAPP pH_7.5 | Root | PM | **Metabolite** | PDR8 (ABCG36) | S45 | 100 | **(39)** |
| AT1G15210 | DAPP pH_7.5 | Root | PM | **Metabolite** | PDR7 (ABCG35) | S43 | 100 | **(102)** |
| AT4G13510 | DAPP pH_7.5 | Root | PM | **Ammonium** | AMT1;1 | T460 | 100 | **(45, 101)** |
| AT3G24300 | DAPP pH_7.5 | Root | PM | **Ammonium** | AMT1;3 | T464 | 100 | **(45, 94, 101)** |
| AT4G30190 | DAPP pH_4.5 | Shoot | PM | **Proton** | AHA2 , PMA2, H(+)-ATPase 2 | T980 | 100 | **(97-100), T948** |
| AT1G59870 | DAPP pH_7.5 | Shoot | PM | **Metabolite** | PDR8 (ABCG36) | S841 | 100 | **(39)** |
| AT4G13510 | DAPP pH_7.5 | Shoot | PM | **Ammonium** | AMT1;1 | T460 | 100 | **(45, 101)** |

**Reference-**

39. Aryal, B., Xia, J., Hu, Z., Stumpe, M., Tsering, T., Liu, J., Huynh, J., Fukao, Y., Glockner, N., Huang, H. Y., Sancho-Andres, G., Pakula, K., Ziegler, J., Gorzolka, K., Zwiewka, M., Nodzynski, T., Harter, K., Sanchez-Rodriguez, C., Jasinski, M., Rosahl, S., and Geisler, M. M. (2023) An LRR receptor kinase controls ABC transporter substrate preferences during plant growth-defense decisions. *Curr Biol* 33, 2008-2023 e2008

43. Sun, D., Fang, X., Xiao, C., Ma, Z., Huang, X., Su, J., Li, J., Wang, J., Wang, S., and Luan, S. (2021) Kinase SnRK1. 1 regulates nitrate channel SLAH3 engaged in nitrate-dependent alleviation of ammonium toxicity. *Plant Physiology* 186, 731-749

44. Lehmann, J., Jørgensen, M. E., Fratz, S., Müller, H. M., Kusch, J., Scherzer, S., Navarro-Retamal, C., Mayer, D., Böhm, J., and Konrad, K. R. (2021) Acidosis-induced activation of anion channel SLAH3 in the flooding-related stress response of Arabidopsis. *Current Biology* 31, 3575-3585. e3579

45. Straub, T., Ludewig, U., and Neuhäuser, B. (2017) The kinase CIPK23 inhibits ammonium transport in Arabidopsis thaliana. *Plant Cell* 29, 409-422

91. Wang, X., Bian, Y., Cheng, K., Gu, L.-F., Ye, M., Zou, H., Sun, S. S.-M., and He, J.-X. (2013) A large-scale protein phosphorylation analysis reveals novel phosphorylation motifs and phosphoregulatory networks in Arabidopsis. *Journal of Proteomics* 78, 486-498

92. Ladwig, F., Dahlke, R. I., Stührwohldt, N., Hartmann, J., Harter, K., and Sauter, M. (2015) Phytosulfokine regulates growth in Arabidopsis through a response module at the plasma membrane that includes CYCLIC NUCLEOTIDE-GATED CHANNEL17, H+-ATPase, and BAK1. *Plant Cell* 27, 1718-1729

93. Roitinger, E., Hofer, M., Köcher, T., Pichler, P., Novatchkova, M., Yang, J., Schlögelhofer, P., and Mechtler, K. (2015) Quantitative Phosphoproteomics of the Ataxia Telangiectasia-Mutated (ATM) and Ataxia Telangiectasia-Mutated and Rad3-related (ATR) Dependent DNA Damage Response in Arabidopsis thaliana*[S]. *Molecular & Cellular Proteomics* 14, 556-571

94. Menz, J., Li, Z., Schulze, W. X., and Ludewig, U. (2016) Early nitrogen‐deprivation responses in Arabidopsis roots reveal distinct differences on transcriptome and (phospho‐) proteome levels between nitrate and ammonium nutrition. *The Plant Journal* 88, 717-734

95. Bhaskara, G. B., Wen, T.-N., Nguyen, T. T., and Verslues, P. E. (2017) Protein phosphatase 2Cs and microtubule-associated stress protein 1 control microtubule stability, plant growth, and drought response. *Plant Cell* 29, 169-191

96. Nimmanon, T. (2016) ﻿ Post− translational mechanisms of the ZIP family of zinc channels. Cardiff University

97. Niittyla, T., Fuglsang, A. T., Palmgren, M. G., Frommer, W. B., and Schulze, W. X. (2007) Temporal analysis of sucrose-induced phosphorylation changes in plasma membrane proteins of Arabidopsis. *Molecular & Cellular Proteomics* 6, 1711-1726

98. Nühse, T. S., Bottrill, A. R., Jones, A. M., and Peck, S. C. (2007) Quantitative phosphoproteomic analysis of plasma membrane proteins reveals regulatory mechanisms of plant innate immune responses. *The Plant Journal* 51, 931-940

99. E. Stecker, K., Minkoff, B. B., and Sussman, M. R. (2014) Phosphoproteomic analyses reveal early signaling events in the osmotic stress response. *Plant Physiology* 165, 1171-1187

100. Chen, Y., Hoehenwarter, W., and Weckwerth, W. (2010) Comparative analysis of phytohormone‐responsive phosphoproteins in Arabidopsis thaliana using TiO2‐phosphopeptide enrichment and mass accuracy precursor alignment. *Plant Journal* 63, 1-17

101. Lanquar, V., Loqué, D., Hörmann, F., Yuan, L., Bohner, A., Engelsberger, W. R., Lalonde, S., Schulze, W. X., von Wirén, N., and Frommer, W. B. (2009) Feedback inhibition of ammonium uptake by a phospho-dependent allosteric mechanism in Arabidopsis. *The Plant Cell* 21, 3610-3622

102. Zhang, H., Zhou, H., Berke, L., Heck, A. J., Mohammed, S., Scheres, B., and Menke, F. L. (2013) Quantitative phosphoproteomics after auxin-stimulated lateral root induction identifies an SNX1 protein phosphorylation site required for growth. *Molecular & Cellular Proteomics* 12, 1158-1169
