# Supplementary material for: Protein Phosphorylation Orchestrates Acclimations of Arabidopsis Plants to Environmental pH
Source: Mol Cell Proteomics. 2023 Nov 23;23(1):100685. doi: 10.1016/j.mcpro.2023.100685 (PMC10837763; doi:10.1016/j.mcpro.2023.100685)
Supplement: Supplemental Table S2 [file mmc6.docx]

Supplementary Table S2. Overlap of DEPs/DPPs identified in this study with previously reported differentially expressed genes between plants grown under control conditions and plants grown on acidic or alkaline conditions. Data are mined from Lager et al. (2010) (11) and Bailey et al. (2022) (12)

| S. No. | Accession | Gene |
| --- | --- | --- |
| 1 | AT1G03870 | FLA9, FASCICLIN-like arabinoogalactan 9 |
| 2 | AT1G11330 | S-locus lectin protein kinase family protein |
| 3 | AT1G17190 | ATGSTU26, GSTU26, glutathione S-transferase tau 26 |
| 4 | AT1G20450 | ERD10, LTI29, LTI45, Dehydrin family protein |
| 5 | AT1G64160 | Disease resistance-responsive (dirigent-like protein) family protein |
| 6 | AT1G70170 | MMP, matrix metalloproteinase |
| 7 | AT2G17500 | Auxin efflux carrier family protein |
| 8 | AT2G23120 | Late embryogenesis abundant protein, group 6 |
| 9 | AT2G26560 | PLA IIA, PLA2A, PLP2, PLP2, phospholipase A 2A |
| 10 | AT2G41100 | ATCAL4, TCH3, Calcium-binding EF hand family protein |
| 11 | AT2G41380 | S-adenosyl-L-methionine-dependent methyltransferases superfamily protein |
| 12 | AT3G05900 | neurofilament protein-related |
| 13 | AT3G28510 | P-loop containing nucleoside triphosphate hydrolases superfamily protein |
| 14 | AT3G55740 | ATPROT2, PROT2, proline transporter 2 |
| 15 | AT4G08770 | Peroxidase superfamily protein |
| 16 | AT4G15530 | PPDK, pyruvate orthophosphate dikinase |
| 17 | AT4G15610 | Uncharacterised protein family (UPF0497) |
| 18 | AT4G20210 | Terpenoid cyclases/Protein prenyltransferases superfamily protein |
| 19 | AT5G06860 | ATPGIP1, PGIP1, polygalacturonase inhibiting protein 1 |
| 20 | AT5G25460 | Protein of unknown function, DUF642 |
| 21 | AT5G44020 | HAD superfamily, subfamily IIIB acid phosphatase |
| 22 | AT5G48010 | THAS, THAS1, thalianol synthase 1 |
| 23 | AT5G52310 | COR78, LTI140, LTI78, RD29A |
| 24 | AT5G64260 | EXL2, EXORDIUM like 2 |
| 25 | AT5G66210 | CPK28, calcium-dependent protein kinase 28…… |
| 26 | AT1G06210 | ENTH/VHS/GAT family protein |
| 27 | AT1G08090 | ATNRT2:1, LIN1, NRT2, NRT2.1, NRT2:1, NRT2;1AT, |
| 28 | AT1G17210 | ATILP1, ILP1, IAP-like protein 1 |
| 29 | AT1G30270 | ATCIPK23, CIPK23, LKS1, SnRK3.23, CBL-interacting protein kinase 23 |
| 30 | AT1G47900 | Plant protein of unknown function (DUF869) |
| 31 | AT1G49410 | TOM6, translocase of the outer mitochondrial membrane 6 |
| 32 | AT1G50660 | unknown protein |
| 33 | AT1G54170 | CID3, CTC-interacting domain 3 |
| 34 | AT1G55500 | ECT4, evolutionarily conserved C-terminal region 4 |
| 35 | AT1G59870 | ATPDR8, PDR8, PEN3, |
| 36 | AT1G61560 | ATMLO6, MLO6, Seven transmembrane MLO family protein |
| 37 | AT1G72790 | hydroxyproline-rich glycoprotein family protein |
| 38 | AT1G76810 | eukaryotic translation initiation factor 2 (eIF-2) family protein |
| 39 | AT2G16900 | Arabidopsis phospholipase-like protein (PEARLI 4) family |
| 40 | AT2G17560 | HMGB4, NFD04, NFD4, high mobility group B4 |
| 41 | AT2G17870 | ATCSP3, CSP3, cold shock domain protein 3 |
| 42 | AT2G19640 | ASHR2, SDG39, ASH1-related protein 2 |
| 43 | AT2G20960 | pEARLI4, Arabidopsis phospholipase-like protein (PEARLI 4) family |
| 44 | AT2G26900 | Sodium Bile acid symporter family |
| 45 | AT2G31570 | ATGPX2, GPX2, glutathione peroxidase 2 |
| 46 | AT2G36460 | Aldolase superfamily protein |
| 47 | AT2G36620 | RPL24A, ribosomal protein L24 |
| 48 | AT2G41810 | Protein of unknown function, DUF642 |
| 49 | AT2G43680 | IQD14, IQ-domain 14 |
| 50 | AT2G44430 | DNA-binding bromodomain-containing protein |
| 51 | AT2G45070 | SEC61 BETA, Preprotein translocase Sec, Sec61-beta subunit protein |
| 52 | AT3G01460 | ATMBD9, MBD9, methyl-CPG-binding domain 9 |
| 53 | AT3G08510 | ATPLC2, PLC2, phospholipase C 2 |
| 54 | AT3G09000 | proline-rich family protein |
| 55 | AT3G13920 | EIF4A1, RH4, TIF4A1, eukaryotic translation initiation factor 4A1 |
| 56 | AT3G19615 | unknown protein |
| 57 | AT3G19930 | ATSTP4, STP4, sugar transporter 4 |
| 58 | AT3G22160 | VQ motif-containing protein |
| 59 | AT3G25190 | Vacuolar iron transporter (VIT) VTL5 |
| 60 | AT3G26400 | EIF4B1, eukaryotic translation initiation factor 4B1 |
| 61 | AT3G53500 | RSZ32, RNA-binding (RRM/RBD/RNP motifs) family |
| 62 | AT3G56990 | EDA7, embryo sac development arrest 7 |
| 63 | AT3G60240 | CUM2, EIF4G, eukaryotic translation initiation factor 4G |
| 64 | AT3G60320 | Protein of unknown function (DUF630 and DUF632) |
| 65 | AT3G61370 | Protein of unknown function (DUF3245) |
| 66 | AT3G61860 | ATRSP31, RSP31, RNA-binding (RRM/RBD/RNP motifs) family protein |
| 67 | AT4G01090 | Protein of unknown function (DUF3133) |
| 68 | AT4G13510 | AMT1;1, ATAMT1, ATAMT1;1, ammonium transporter 1;1 |
| 69 | AT4G14990 | Topoisomerase II-associated protein PAT1 |
| 70 | AT4G20980 | Eukaryotic translation initiation factor 3 subunit 7 (eIF-3) |
| 71 | AT4G21180 | ATERDJ2B, DnaJ / Sec63 Brl domains-containing protein |
| 72 | AT4G25340 | ATFKBP53, FKBP53, FK506 BINDING PROTEIN 53 |
| 73 | AT4G26050 | PIRL8, plant intracellular ras group-related LRR 8 |
| 74 | AT4G30010 | unknown protein. |
| 75 | AT4G30440 | GAE1, UDP-D-glucuronate 4-epimerase 1 |
| 76 | AT4G31700 | RPS6, RPS6A, ribosomal protein S6 |
| 77 | AT4G36210 | Protein of unknown function (DUF726) |
| 78 | AT4G38470 | ACT-like protein tyrosine kinase family protein |
| 79 | AT5G22650 | ATHD2, ATHD2B, HD2, HD2B, HDA4, HDT02, HDT2, histone deacetylase 2B |
| 80 | AT5G23080 | TGH, SWAP (Suppressor-of-White-APricot)/surp domain-containing protein |
| 81 | AT5G24290 | Vacuolar iron transporter (VIT) family protein |
| 82 | AT5G38200 | Class I glutamine amidotransferase-like superfamily protein |
| 83 | AT5G45510 | Leucine-rich repeat (LRR) family protein |
| 84 | AT5G47210 | Hyaluronan / mRNA binding family |
| 85 | AT5G48430 | Eukaryotic aspartyl protease family protein |
| 86 | AT5G52040 | ATRSP41, RNA-binding (RRM/RBD/RNP motifs) family protein |
| 87 | AT5G56980 | unknown protein |

Table S2. Orange indicate overlap between Lager et al. (2010) (11) and this study. Purple indicates overlap between Bailey et al. (2022) (12) and this study.

Reference-

11. Lager, I., Andréasson, O., Dunbar, T. L., Andreasson, E., Escobar, M. A., and Rasmusson, A. G. (2010) Changes in external pH rapidly alter plant gene expression and modulate auxin and elicitor responses. *Plant, Cell & Environment* 33, 1513-1528

12. Bailey, M., Hsieh, E.-J., Tsai, H.-H., Ravindran, A., and Schmidt, W. (2022) Alkalinity modulates a unique suite of genes to recalibrate growth and pH homeostasis. *Frontiers in Plant Science*, 2022.2012. 2012.520164
